# A novel and apparent *de novo ALAS2* missense variant associated with congenital sideroblastic anemia

**DOI:** 10.3389/fped.2024.1411676

**Published:** 2024-08-29

**Authors:** Jianling Cai, Tianming Liu, Yuxuan Huang, Hongxing Chen, Meidie Yu, Dongqing Zhang, Zhanqin Huang

**Affiliations:** ^1^ Department of Pediatrics, The First Affiliated Hospital of Shantou University Medical College, Shantou, Guangdong, China; ^2^ Department of Laboratory Medicine, The First Affiliated Hospital of Shantou University Medical College, Shantou, Guangdong, China; ^3^ Department of Clinical Medicine, Shantou University Medical College, Shantou, Guangdong, China; ^4^ Department of Pharmacology, Shantou University Medical College, Shantou, Guangdong, China

**Keywords:** congenital, sideroblastic anemia, *ALAS2*, in silico analysis, pyridoxine

## Abstract

**Background:**

Congenital sideroblastic anemia (CSA) constitutes a group of inherited erythropoietic disorders. Some affect mainly or exclusively erythroid cells; other syndromic forms occur within multisystem disorders with extensive nonhematopoietic manifestations. In this study, we have performed clinical and molecular investigations on a 10-year-old boy suspected of having CSA.

**Methods:**

Routine blood examination, peripheral blood and bone marrow smears, and serum iron tests were performed. Gene mutation analysis was conducted using whole-exome sequencing (WES) and the results were confirmed using Sanger sequencing. Furthermore, the functional impact of the identified variant was assessed/predicted with bioinformatics methods.

**Results:**

The patient presented with severe microcytic anemia (hemoglobin, 50 g/L), iron overload and ring sideroblasts in the bone marrow. Moreover, WES revealed the presence of a hemizygous missense variant in *ALAS2* (c.1102C > T), changing an encoded arginine to tryptophan (p. Arg368Trp). This variant was verified via Sanger sequencing, and neither of the parents carried this variant, which was suspected to be a *de novo* variant. Using *in silico* analysis with four different software programs, the variant was predicted to be harmful. PyMol and LigPlot software showed that the p. Arg368Trp variant may result in changes in hydrogen bonds. The patient was treated with vitamin B6 combined with deferasirox. After 6 months, the hemoglobin increased to 99 g/L and the serum ferritin decreased significantly.

**Conclusion:**

We report a novel pathogenic variant in the *ALAS2* gene (c.1102C > T:p. Arg368Trp), which caused CSA in a 10-year-old boy. Mutational analysis is important in patients with CSA when family history data are unavailable. Anemia due to the *ALAS2* Arg368Trp variant responds to pyridoxine supplements.

## 1 Introduction

Sideroblastic anemias (SAs) are a heterogeneous group of disorders characterized by the presence of abnormal erythroid precursors with perinuclear mitochondrial iron deposition in the bone marrow. Although their prevalence has not been defined, they are uncommon and occur in congenital and acquired forms (1–3). In general, there are two clinical manifestations of congenital sideroblastic anemia (CSA). One form affects mainly or exclusively erythroid cells, which is non-syndromic with only presenting hematopoietic disorders. Another form has associated syndromic features, which involves multiple system disorders. Nevertheless, many of CSA have mutations in genes involved in heme biosynthesis, iron-sulfur cluster biosynthesis, and mitochondrial protein synthesis. To date, over 10 pathogenic genes associated with CSA have been reported, including *ALAS2*, *SLC25A38*, *GLRX5*, *HSPA9*, *HSCB*, *ABCB7*, *PUS1*, *YARS2*, *LARS2*, *TRNT1*, *MT-ATP6*, *NDUFB11*, and *SLC19A2* (4–7)_._ X-linked sideroblastic anemia (XLSA), which is the most common form of non-syndromic CSA. It was caused by variants in the erythroid-specific 5-aminolevulinate synthase (*ALAS2*) gene (1). Here, we report the case of a patient presenting with characteristics of CSA in whom we identified a novel pathogenic variant in the *ALAS2* gene.

## 2 Materials and methods

### 2.1 Patient

A 10-year-old boy was admitted to another hospital with a 3-year history of chronic anemia. Due to born in the area, Guangdong province in PRC, with high incidence of thalassemia, this patient was conducted with the thalassemia genetic testing. The result was normal, with no large deletion/duplications in the alpha-globin gene cluster or pathogenic variants in the beta-globin gene. Bone marrow examination showed erythroid-predominant trilineage hematopoiesis. Although his condition improved after several red blood cell transfusions, the diagnosis was unclear. However, when symptoms worsened after 3 months, he visited our hospital for further evaluation.

### 2.2 Routine examination

Samples were collected from the patient and his parents. Blood tests were performed using an LH 780 automated hematology analyzer (Beckman Coulter, CA, USA). Iron metabolism tests, including serum iron and ferritin, were performed using a 7,600 Fully Automated Biochemical Analyzer (Hitachi, Ltd., Tokyo, Japan). Bone marrow smears were obtained, and Perls’ staining was performed.

### 2.3 Whole-exome sequencing

Genomic DNA was analyzed using whole-exome sequencing (WES). To this end, 2 ml of peripheral blood was collected from the patient and his parents. Genomic DNA was isolated from leukocytes using an QIAamp DNA Blood Midi Kit (Qiagen, Hilden, Germany) according to the manufacturer's instructions. Exomes were captured using a TruSight One Sequencing Panel (Illumina Inc., USA), and sequencing was conducted using an Illumina HiSeq (Illumina Inc., USA). The results were aligned with the University of California, Santa Cruz Human Genome Assembly 19 (UCSC.hg19; http://genome.ucsc.edu/) reference sequence. Variants were filtered using several common variant databases, including the 1,000 genome database, dbSNP database, ClinVar database, Polymorphism Phenotyping v2 software, and Sorting Intolerant from Tolerant algorithm. The pathogenicity of the filtered candidate variants was determined according to the ACMG (American College of Medical Genetics and Genomics) guidelines (8). The ACMG classifications of variations include pathogenic, likely pathogenic, uncertain significance, likely benign, and benign. Based on the patient's clinical history, variants in candidate genes linked to CSA were prioritized.

### 2.4 Validation of the candidate variant by sanger sequencing

The variant that most likely contributed to the disease was verified by Sanger sequencing. Briefly, the selected *ALAS2* gene region, including the Arg368Trp variant, was amplified by Polymerase chain reactions (PCR) and sequenced by Sanger sequencing. PCR amplification was performed using an ABI system. The PCR products were loaded onto an ABI3500XL. The results were analyzed using Variant Reporter v 1.1 software and Sequencing Analysis 5.2 software. Primer pairs were designed using the PrimerQuest Tool (http://sg.idtdna.com/Primerquest/Home/Index). The primers used to confirm the *ALAS2* Arg368Trp variant were as follows:

Forward primer: 5′AGCTGGGGAAGGGTTATGAT3′

Reverse primer: 5′GGGAGGAGGCAGAAAAGAAT3′

### 2.5 Mutation analysis for *ALAS2*

Bioinformatics software was used to analyze and predict the effect of the missense variant (p. Arg368Trp) on *ALAS2* functioning. For this purpose, the PolyPhen-2 (http://genetics.bwh.harvard.edu/pph2/), SIFT (http://sift.bii.a-star.edu.sg, FATHMM (http://fathmm.biocompute.org.uk), and Mutation Assessor (http://mutationassessor.org) bioinformatics programs were selected. To study the effect of c.1102C > T on the three-dimensional (3D) crystal structure of the protein, computational modeling was performed for normal and mutant *ALAS2* molecules using the online software program Swiss-model (https://swissmodel.expasy.org/). The template protein structure was obtained from the Protein Data Bank (https://www.rcsb.org/pdb/home/home.do). PyMol and LigPlot software were employed to perform a comparative analysis, make necessary modifications, and visualize the molecular structures of both the wild-type and variant forms of the ALAS2 protein.

## 3 Results

### 3.1 Clinical features

**Figure 1:**
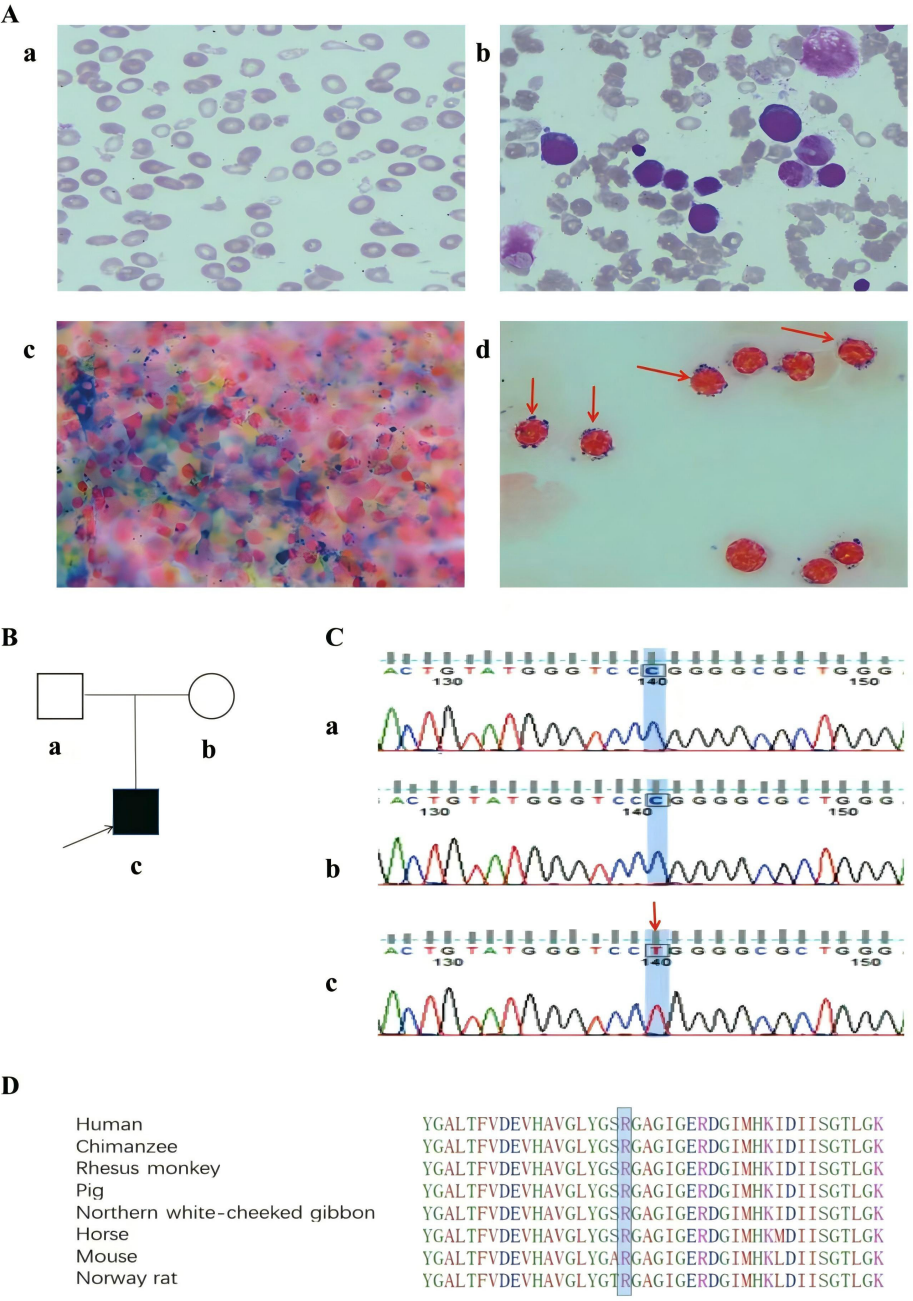
**(A)** Peripheral blood and bone marrow morphology of the patient. (a) Peripheral blood smear showing anisopoikilocytosis, microcytosis, and hypochromia (Wright-Giemsa stain, ×1000). (b) Bone marrow smear showing erythroid hyperplasia without dysplasia (Wright-Giemsa stain, ×1000). (c, d) Perls' staining showing increased iron stores with the presence of ring sideroblasts (Perls' stain, ×1000); the red arrow indicates ring sideroblasts. **(B)** Pedigree of the family. (a) Patient's father, (b) Patient's mother, (c) Patient. **(C)** Genetic analysis of the family: A novel variant in exon 8 (c.1102C>T) of the ALAS2 gene in the patient. The red arrow indicates point variant. (a) Patient's father, (b) Patient's mother, (c) Patient; **(D)** Bioinformatics conservative analysis among humans, mice, Norway rats, chimpanzees, rhesus monkeys, horses, pigs, and northern white-cheeked gibbons at the variant position.

Clinical examination was normal except for severe anemia and splenomegaly. Abdominal ultrasonographic findings revealed splenomegaly (spleen length: 148 mm, thickness: 51 mm, subcostal: 38 mm). The complete blood count was as follows: white blood cells (WBC), 4.2 × 10^9^/L (normal value, 4.00–10.00 × 10^9^/l); red blood cells (RBC), 2.51 × 10^12^/l (normal value, 4.20–5.70 × 10^12^/L); Hemoglobin (Hb), 50 g/L (normal value, 118–156 g/L); mean corpuscular volume (MCV), 65.3 fL (normal value, 77.0–92.0 fL); mean corpuscular hemoglobin (MCH), 19.9 pg (normal value, 25.0–34.0 pg); mean corpuscular hemoglobin concentration (MCHC), 305 g/L (normal value, 310–355 g/L); red cell distribution width (RDW), 32.0% (normal value, 11.5–14.5%); reticulocyte (RET), 2.53% (normal value, 0.50%–2.50%); platelet (PLT), 170 × 10^9^/L (normal value, 167–453 × 10^9^/L). Tests for iron levels indicated iron overload, with a serum iron level of 40.33 µmol/L (normal value, 9.00–22.00 µmol/L) and a ferritin level of 2,267.0 µg/L (normal value, 6.1–252.5 µg/L). Red cell morphology showed marked microcytosis, a few red cell fragmentations, and anisopoikilocytosis. Bone marrow aspiration revealed erythroid hyperplasia with no dysplastic features. Perls’ staining revealed increased iron stores with the presence of ring sideroblasts in 36% of the developing erythrocytes (Figure 1A).

### 3.2 DNA analysis

WES was performed on the proband, and 13G of raw data was obtained. The proportions of base quality values ≥30 (Q30) were 93.38%. The average sequencing depth for the target region was 136X, with more than 99% of the target sequence reaching up to 20X. A missense variant (c.1102C > T: p. Arg368Trp) in exon 8 of the *ALAS2* gene was identified. Sanger sequencing confirmed that the variant was hemizygous in the proband but absent from his parents (Figures 1B,C). According to the ACMG guidelines, the variant was classified as variant of uncertain significance (PM6 + PM2_supporting + PP4). Based on the amino acid sequence of ALAS2 protein in the PUBMED website, we selected eight species, including humans, mice, Norway rats, chimpanzees, rhesus monkeys, horses, pigs, and northern white-cheeked gibbons, for the homology analysis of ALAS2 protein sequences. We found that *ALAS2* is highly conserved across these species at the variant position (Figure 1D).

### 3.3 Prediction and analysis of online protein structure

**Figure 2:**
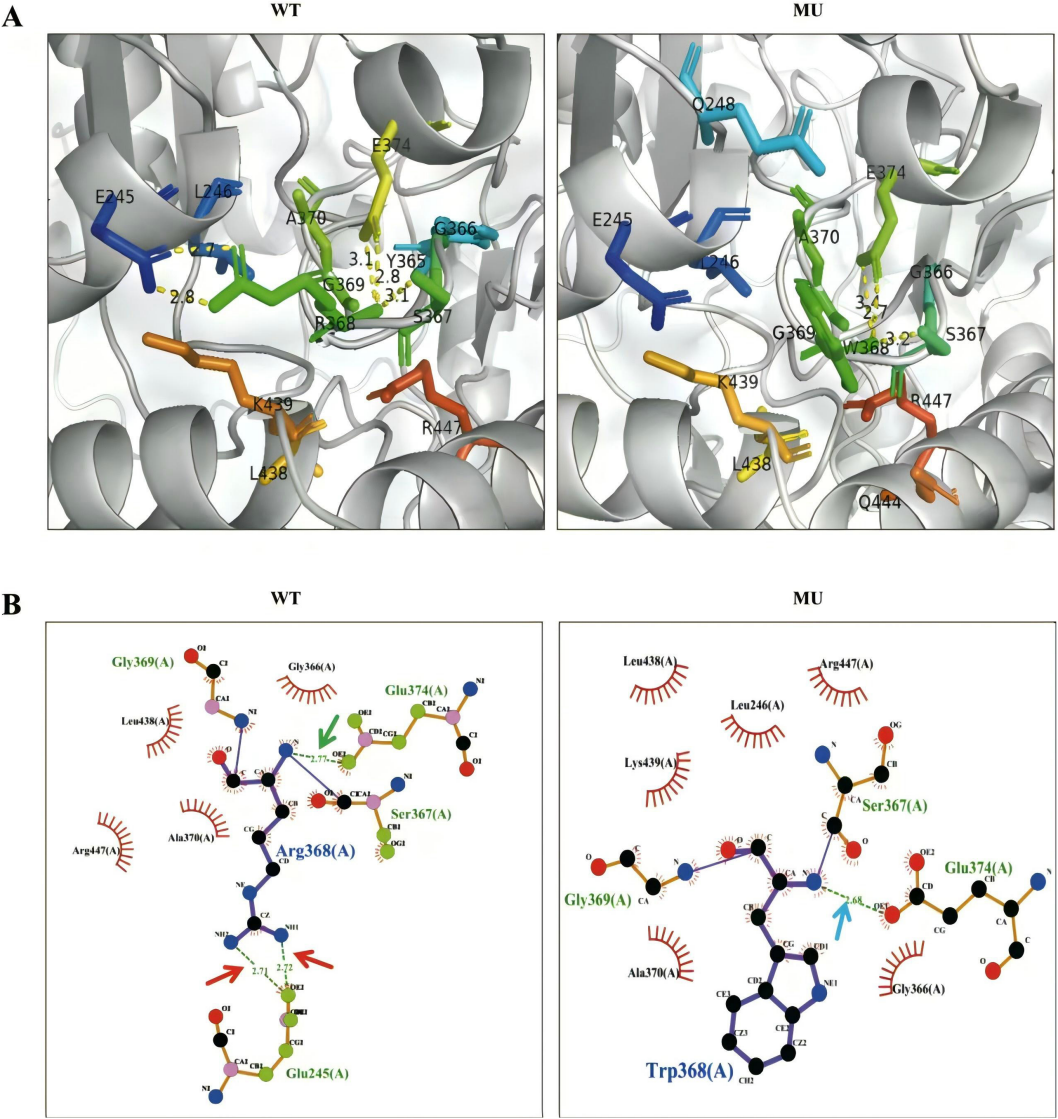
Structural analysis between wild-type (WT) and mutant-type ALAS2 protein. **(A)** PyMol software showed that the Arg368Trp variant disrupted hydrogen bonds with Glu245. The WT ALAS2 protein R368 forms two hydrogen bonds with E245 and two hydrogen bonds with E374, whereas the ALAS2 mutant protein W368 forms only two hydrogen bonds with E374. **(B)** LigPlot software showed that the Arg368Trp variant disrupted hydrogen bonds with both Glu245 and Glu374. The WT ALAS2 protein Arg368 forms two hydrogen bonds with Glu245 (red arrow) and one hydrogen bond with Glu374 (green arrow), whereas the ALAS2 mutant protein Trp368 forms only one hydrogen bond with Gly366 (blue arrow).

Polyphen-2, SIFT, FATHMM, and MutationAssessor were used to predict the pathogenicity of the ALAS2 variant (c.1102C > T), as shown in Table 1. PyMol and LigPlot were used to predict changes in hydrogen bonds when tryptophan was substituted with arginine at position 368 of ALAS2 protein. Arg368 is involved in the formation of hydrogen bonds with glutamic acid (Glu) residues at positions 245 and 374. PyMol analyses indicated that the Arg368Trp variant had no effect on hydrogen bonds with Glu374, but it disrupted the hydrogen bonds with Glu245. LigPlot analyses indicated that the variant disrupted hydrogen bonds with both Glu245 and Glu374 (Figure 2).

**Table 1:** In silico analysis of ALAS2 variant (c.1102C>T).

Tool	Score	Result
Polyphen-2	0.995	Damaging
SIFT	0	Deleterious
FATHMM	−6.37	Deleterious
MutationAssessor	2.665	Disease associate

Predictions for missense substitutions and scores are indicated for software analysis. Polyphen-2 scores: 0–0.14, benign; 0.15–0.84, possibly damaging; and 0.85–1, damaging. SIFT scores: >0.05 indicative of tolerated substitution, ≤0.05 indicative of not tolerated or deleterious substitution. FATHMM score < −1.5 indicative of a deleterious substitution. MutationAssessor scores: −5.76–5.37, score <1.938 is polymorphism associated and score >1.938 is disease-associated.

### 3.4 Case follow-up

The patient was treated with vitamin B6 (60 mg/day) and deferasirox (250 mg/day). He responded well, with a gradual increase in Hb and a gradual decrease in ferritin. After 6 months, his Hb level increased to 99 g/L and ferritin level decreased to 924.3 µg/L. However, the microscopic hypochromic features of erythrocytes remained, which showed little improvement (Table 2). Abdominal ultrasonographic findings showed a considerable reduction in the size of the spleen (spleen length: 137 mm, thickness: 42 mm, subcostal: 12 mm).

**Table 2:** Hematological findings before and after treatment with vitamin B6.

	Normal values	At diagnosis	At 1 month	At 3 months	At 6 months
RBC (×10^12^/L)	4.20–5.70	2.51	3.21	3.89	4.58
Hb (g/L)	118–156	50	77	81	99
MCV (fL)	77.0–92.0	65.3	73.2	66.3	68.6
MCH (pg)	25.0–34.0	19.9	24	20.8	21.6
MCHC (g/L)	310–355	305	328	314	315
Ferritin (µg/L)	6.1–252.5	2,267.0	1,732.0	1,294.1	924.3

## 4 Discussion

Ring sideroblasts in the bone marrow are the hallmark of sideroblastic anemias. The child presented with microcytic anemia, high serum ferritin levels, and notably ring sideroblasts in the bone marrow. The possibility of CSA was considered based on young age at disease onset, spleen enlargement, and lack of a history of exposure to drugs or toxins. However, the parents of the child were healthy. Currently, only molecular testing can be performed to determine the cause. Analysis using WES revealed a hemizygous missense variant in exon 8 of the *ALAS2* gene (c.1102C > T:p. Arg368Trp), which has not been previously reported. Because the variant was classified as of unknown significance according to ACMG, the functional impact of the variant was further analyzed using bioinformatics methods. Multiple sequence alignment of ALAS2 from several species revealed that Arg368 was located within a highly conserved region. The p. Arg368Trp variant was predicted to be harmful by four separate bioinformatic software programs. In addition, the molecular visualization system revealed the changes in hydrogen bonds. Hydrogen bonds are essential interatomic bonds to protein molecules because they maintain the shape of proteins. Generally, more hydrogen bonds make the protein more stable (9). When tryptophan is substituted for arginine at position 368, the hydrogen bonds are reduced, potentially leading to structural and functional alterations in the protein. Consequently, such a substitution is considered damaging. We have not formally validated the Arg368Trp variant as pathogenic with a functional assay. However, based on our in silico analysis, combined with the significant therapeutic improvement of the patient after vitamin B6 supplementation, which is typical of XLSA, we concluded that the Arg368Trp variant is likely pathogenic.

Because *ALAS2* is located on the X chromosome, CSA caused by the mutation of the *ALAS2* exhibits an X-linked inheritance pattern (10). Most males carrying the XLSA allele will express the disease, and many females carrying the allele will do as well (11, 12). In our case, although the child carried a pathogenic variant of the *ALAS2* gene, sequencing data showed that the parents did not carry this variant; thus, the variant is likely be *de novo*. An *et al*. reported that 3 of 9 patients with XLSA had *de novo* mutations, suggesting that the disease has a somewhat high *de novo* mutation frequency (13). To date, approximately 119 pathogenic variants in the *ALAS2* have been reported in patients with XLSA, and the mutations are mainly attributed to the hypermutability of CpG dinucleotides, which also contributes to the relative frequency of *de novo* mutations in this disease (2, 14).

ALAS2 is the first enzyme in the heme biosynthesis pathway in erythroid cells. It uses pyridoxal phosphate (PLP) as a coenzyme to catalyze the synthesis of glycine and succinyl-coA into delta aminolevulinic acid (ALA) (15). ALAS2 consists of 587 amino acids, comprising an N-terminal region (aa 1–142), a conserved catalytic core (aa 143–544), and a C-terminal extension (aa 545–587) (16). Researchers reported that most of the pathogenic variants are located in exon 5 and exon 9, the latter containing the PLP-binding binding site, lysine 391. Generally, patients with variants close to the PLP-binding site respond favourably to pyridoxine therapy, whereas those with variants distant from the site are refractory to treatment (17–19). In our patient, the Arg368Trp variant being located in the catalytic domain and near the PLP-binding site along with the weakened hydrogen bonds would explain the favorable response to pyridoxine supplements.

Blood transfusion is currently the primary treatment in patients with pyridoxine-resistant severe CSA. However, long-term transfusion is associated with the risk of transfusion-related complications and iron overload, which can lead to organ dysfunction (20, 21). Hematopoietic stem cell transplantation is the only curative option, but there are challenges, such as graft-versus-host disease (GVHD) (22). Luspatercept is a specific activin receptor fusion protein that acts as a ligand trap to neutralize negative regulators of late-stage erythropoiesis and is mainly used to manage and treat anemia in myelodysplastic syndrome, primary myelofibrosis, and beta-thalassemia (23, 24). Recently, luspatercept has been used to treat CSA. Van *et al*. reported that in a transfusion-dependent 51-year-old man with XLSA, the use of luspatercept led to a 30% increase in Hb levels and a decreased need for transfusion, suggesting its efficacy in the treatment of CSA. However, more clinical trials are needed to validate this approach (25).

In summary, we report a novel pathogenic variant in the *ALAS2* gene (c.1102C > T: p. Arg368Trp), causing XLSA in a 10-year-old boy and expanding the spectrum of ALAS2 variants. The patient responded well to pyridoxine supplementation. Rapid and accurate diagnosis led to effective treatment and a favorable clinical course, thereby reducing long-term complications.

## Data Availability

The raw sequence data reported in this paper have been deposited in the Genome Sequence Archive in National Genomics Data Center, China National Center for Bioinformation/Beijing Institute of Genomics, Chinese Academy of Sciences (https://bigd.big.ac.cn/). After publication of study findings, the data will be available for others to request. The research team will provide accession number of the database once the data are approved to be shared with others. The proposal with description of study objectives will be needed for evaluation of the reasonability to request for the data. The corresponding authors and the Ethical Committee of the First Affiliated Hospital of Shantou University Medical College will make a decision based on these materials.
